# A Retrospective Analysis of Balance Function After Unilateral Total Knee Arthroplasty in Patients With Bilateral Knee Osteoarthritis

**DOI:** 10.7759/cureus.112196

**Published:** 2026-07-07

**Authors:** Venkata Vinay Atluri, Kiminori Yukata, Yutaka Suetomi, Atsunori Tokushige, Hiroshi Fujii, Takashi Sakai

**Affiliations:** 1 Department of Orthopaedic Surgery, Ogori Daiichi General Hospital, Yamaguchi, JPN; 2 Department of Orthopaedic Surgery, Yamaguchi University Graduate School of Medicine, Ube, JPN

**Keywords:** balance function, one-leg standing time, osteoarthritis knee, timed up and go test, total knee arthroplasty (tka)

## Abstract

Purpose: Total knee arthroplasty (TKA) is widely recognized for its efficacy in reducing pain and improving both static and dynamic balance in individuals with end-stage knee osteoarthritis (OA). However, the sustainability of these improvements after unilateral TKA in patients with bilateral knee OA remains unclear.

Methods: This retrospective study analyzed the medical records of patients with bilateral knee OA (Kellgren-Lawrence grade ≥3) who underwent unilateral TKA of the more symptomatic knee and did not undergo contralateral TKA within one year. Balance function was assessed using the timed up and go (TUG) test and the one-leg standing (OLS) test at three time points: preoperatively, six months postoperatively, and at the final follow-up (≥1 year postoperatively).

Results: A total of 51 patients were included in the analysis. Both TUG and OLS times showed significant improvement from the preoperative period to six months postoperatively. At the final follow-up, the balance function showed a decline, suggesting a loss of the initial benefits of unilateral TKA.

Conclusion: Unilateral TKA significantly improves balance function in patients with bilateral knee OA. Although these improvements tended to decline over time, the underlying factors could not be determined in this retrospective study. These findings support the importance of longitudinal follow-up and may assist in clinical decision-making regarding staged bilateral TKA.

## Introduction

Knee osteoarthritis (OA) is a highly prevalent and debilitating condition characterized by pain, functional limitations, and progressive physical disability [1,2]. A substantial proportion of individuals with knee OA experience bilateral symptoms [3], with severe involvement reported in up to 19% of cases [4]. Patients with knee OA frequently present with impaired proprioception, muscle weakness, and chronic knee pain [5-7], all of which contribute to compromised postural stability and an increased risk of falls. Postural stability is fundamental for maintaining body control during static and dynamic activities, including walking, transitioning from sitting to standing, and performing daily tasks [8].

Postural stability encompasses both static and dynamic balance. Static balance refers to the ability to maintain equilibrium while stationary, whereas dynamic balance pertains to stability during movement [8]. The one-leg standing (OLS) test is widely utilized to assess postural stability, particularly in older adults [9-11]. Similarly, the timed up and go (TUG) test is a well-established tool for evaluating functional mobility and balance in individuals with knee OA. Among the various performance-based measures used to assess outcomes following total knee arthroplasty (TKA) [12-14], the TUG test is particularly favored by physical therapists due to its reliability and clinical relevance [12].

TKA is a well-established surgical intervention for alleviating pain and restoring physical function in individuals with advanced knee OA [15]. Studies, including that by Cho et al. [16], have demonstrated significant improvements in postural stability, as measured by the OLS test, following TKA. Although unilateral TKA is commonly performed in patients with bilateral knee OA when one knee is more symptomatic, the durability of the functional benefits provided by unilateral surgery before contralateral intervention remains incompletely understood. Previous studies have consistently demonstrated that unilateral TKA improves pain, function, gait, and balance in patients with knee OA during the early postoperative period [15,16]. However, in patients with bilateral knee OA, it remains unclear whether these functional and balance improvements are sustained over time as the untreated contralateral knee continues to progress. Furthermore, the timing at which any decline in function may occur and its potential implications for the timing of contralateral TKA have not been well established. This knowledge gap underscores the need to evaluate the durability of balance improvements following unilateral TKA in patients with bilateral knee OA. By assessing longitudinal changes in balance and functional performance before and after staged bilateral TKA, the present study provides evidence regarding the persistence of unilateral TKA benefits and identifies when these improvements may begin to diminish, thereby informing clinical decision-making and surgical planning.

This study aimed to investigate temporal changes in balance function and physical performance in patients with bilateral knee OA following unilateral TKA, with particular emphasis on the persistence of these improvements before staged contralateral TKA.

## Materials and methods

We conducted a retrospective analysis of medical records from patients treated at our institution between 2011 and 2020. The inclusion criteria encompassed patients diagnosed with bilateral knee OA classified as Kellgren-Lawrence grade ≥3 [17], who had undergone unilateral TKA on the more symptomatic knee and had not undergone contralateral TKA within one year. The more symptomatic knee was determined based on the patient's reported symptoms, pain severity, functional limitation, clinical examination findings, and radiographic assessment, with the decision regarding the operative side made by the treating surgeon in consultation with the patient. Furthermore, eligible patients were required to have undergone TUG and OLS tests at three specified time points: preoperatively, six months postoperatively, and at the final follow-up (≥1 year after unilateral TKA). 

Balance function was assessed using two validated performance-based measures: the TUG test and the OLS test. The TUG test evaluates dynamic balance and functional mobility by measuring the time (in seconds) required for a patient to rise from a standard chair, walk a distance of three meters, turn, return to the chair, and sit down. This test has been widely validated as a reliable indicator of functional mobility in elderly populations [13]. The OLS test assesses static balance by measuring the duration (in seconds) a patient can stand unassisted on one leg with eyes open. Longer durations indicate better postural stability. This test is a strong predictor of fall risk in older adults [18].

Patients were excluded if they had (i) neurological impairments affecting the lower extremities; (ii) previous surgery involving the lower extremities; (iii) symptomatic hip or lumbar spine disorders or other musculoskeletal conditions that could influence gait or balance assessment; or (iv) a postoperative follow-up period of less than one year after primary TKA. The study was approved by the Institutional Ethics Committee (approval number and date: 24-03, May 27, 2024), and all participants gave written informed consent.

All TKA procedures were performed using a standardized medial parapatellar approach under general anesthesia, with patellar resurfacing conducted in all cases. The posterior-stabilized Persona knee prosthesis (Zimmer, Warsaw, USA) was implanted, and the modified gap-balancing technique was employed [19].

Perioperative blood management strategies included the use of a pneumatic tourniquet and intra-articular tranexamic acid injection at arthrotomy closure. Postoperative care followed a standardized institutional rehabilitation protocol comprising multimodal analgesia, pneumatic calf pump application, initiation of continuous passive motion on postoperative day one, daily physiotherapy assessments, and ambulation beginning on postoperative day two. All patients were prescribed the same postoperative rehabilitation protocol. However, compliance with the rehabilitation program, particularly after hospital discharge, was not formally evaluated.

Statistical analysis

Descriptive statistics, including means and standard deviations, were calculated to summarize patient demographics. The Wilcoxon signed-rank test was used to compare the TUG test and OLS time results for non-normally distributed data. A p-value of <0.05 was considered statistically significant.

## Results

A total of 51 patients with complete datasets were included in the final analysis. The mean age of the cohort was 75.6 ± 5.6 years, with 26 patients undergoing left-sided TKA and 25 undergoing right-sided TKA. Additional demographic details are provided in Table 1.

**Table 1:** Demographic characteristics. Data expressed as number, mean ± standard deviation. TUG: Timed Up and Go, OLS: One-Leg Standing, OA: Osteoarthritis, FTA: Femorotibial Angle

Characteristics	
Gender (Male/Female)	13 (25.5%)/38 (74.5%)
Pathology	Primary OA (n=51)
Height (cm)	153.4 ± 7.9
Weight (kg)	61.0 ± 9.5
Body mass index (kg/m^2^)	25.9 ± 2.9
Final follow-up period (months)	25.9 ± 15.7
FTA (degrees)	184.2 ± 9.1

**Figure 1:**
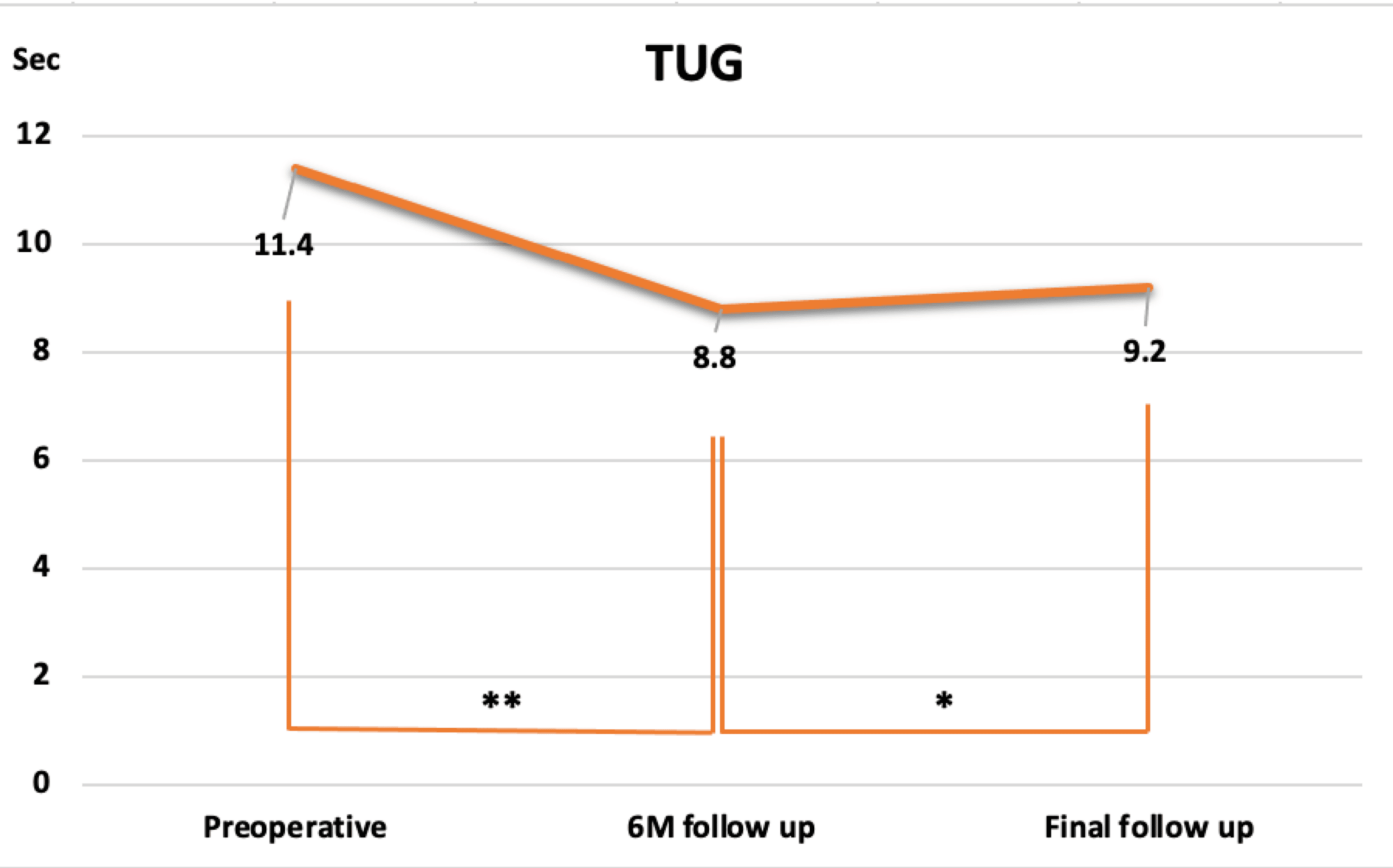
The chart illustrates the changes in the TUG test scores from the preoperative period to the final follow-up after unilateral TKA. ** p <0.001, * p<0.05. TUG: Timed Up and Go

Preoperatively, the mean TUG test time was 11.4 ± 3.7 seconds, which improved significantly to 8.8 ± 2.5 seconds at six months postoperatively (Figure 1; p < 0.05). At the final follow-up, there was a slight but statistically significant increase in the TUG time to 9.2 ± 2.1 seconds (p < 0.05).

**Figure 2:**
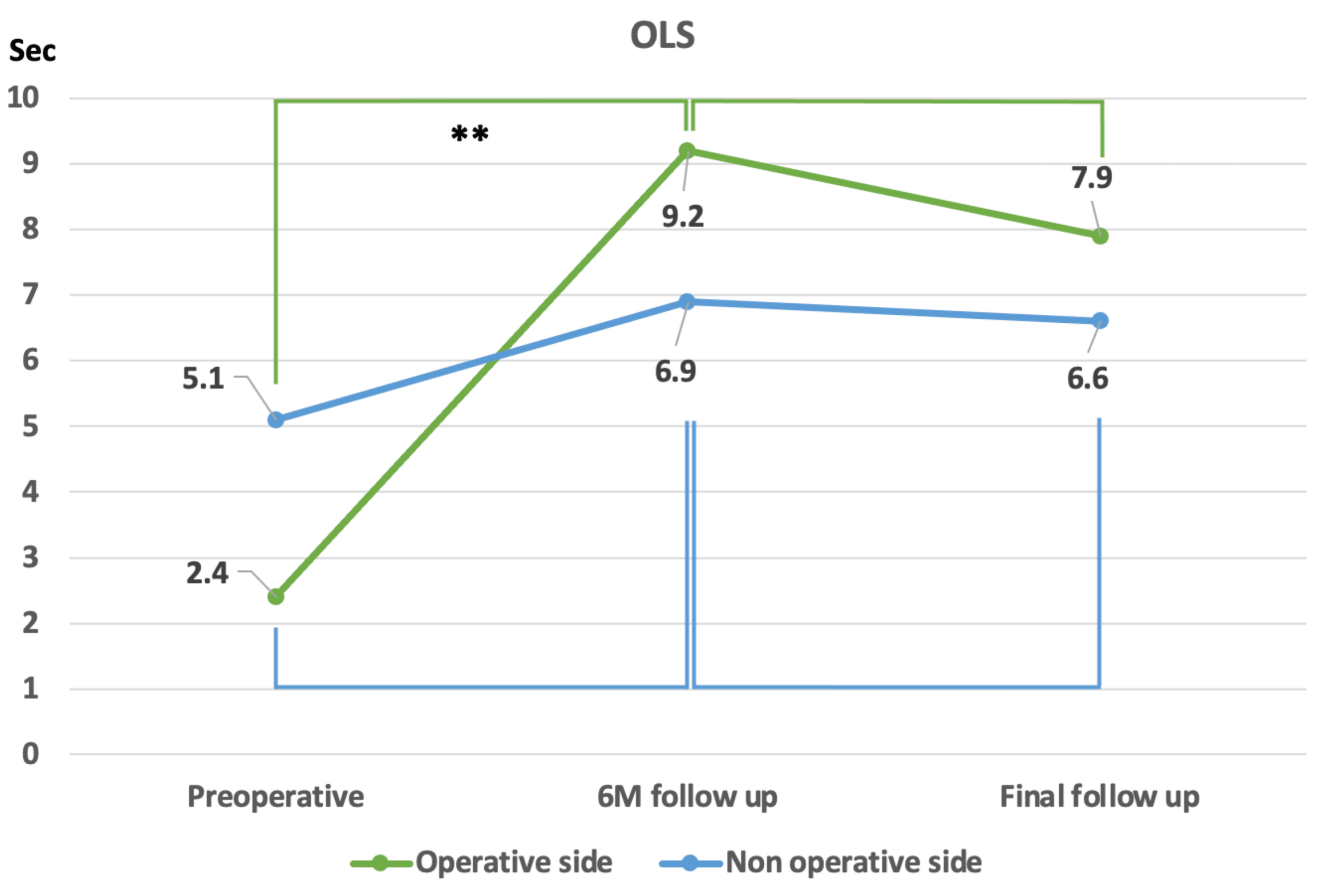
The chart depicts the changes in the OLS test scores for both the operated and non-operated legs from the preoperative period to the final follow-up after unilateral TKA. ** p <0.001. OLS: One-Leg Standing

Regarding the operated limb, the baseline OLS time was 2.4 ± 1.7 seconds, which demonstrated a significant improvement to 9.2 ± 8.4 seconds at six months postoperatively (Figure 2; p < 0.001). However, a slight decrease was observed at the final follow-up, with a mean OLS time of 7.9 ± 8.7 seconds (p = 0.052). On the non-operated side, the baseline OLS time was 5.1 ± 4.7 seconds, increasing to 6.9 ± 7.0 seconds at six months postoperatively, though this change did not achieve statistical significance (p = 0.086). At the final follow-up, a slight decrease was noted, with an average OLS time of 6.6 ± 7.8 seconds (p = 0.465).

## Discussion

The present study demonstrates that unilateral TKA in patients with bilateral knee OA results in significant short-term improvements in both static and dynamic balance, as measured by OLS time and TUG performance. However, a trend toward partial decline in these improvements was observed at final follow-up, suggesting that the durability of these functional gains may be limited in the presence of ongoing contralateral disease.

Patients with knee OA commonly exhibit impaired proprioception, reduced muscle strength, and compromised postural stability, all of which contribute to functional limitations and increased fall risk [5-8]. The OLS test is a widely accepted measure of static balance and has been associated with functional independence and fall risk in elderly populations [9-11]. Similarly, the TUG test is a validated and reliable assessment of functional mobility and dynamic balance in patients with OA [12-14]. In this study, both parameters showed significant improvement at six months postoperatively, consistent with previous literature demonstrating functional recovery following TKA.

The improvement in OLS time observed in the operated limb aligns with previous studies demonstrating enhanced single-limb balance following TKA [16]. However, in our cohort, OLS performance showed a decline at final follow-up, although this did not reach statistical significance. This suggests that while unilateral TKA provides meaningful short-term improvement in static balance, these benefits may diminish over time. 

The role of aging in balance impairment is well established, with advancing age associated with reduced muscle strength, delayed neuromuscular responses, and decreased proprioceptive function [20]. Dynamic balance, as assessed by the TUG test, followed a similar trend, with significant improvement at six months and a slight increase in TUG time at final follow-up. Although overall performance remained improved compared to preoperative values, previous studies suggest that fall risk may persist even after TKA [21]. Established thresholds for TUG performance have been described in the literature [13], indicating that some patients may remain at risk despite postoperative improvement. Additionally, factors such as physical activity levels and comorbidities may continue to influence postoperative outcomes [22].

In the present study, the observed trend toward reduced balance performance over time may reflect the combined effects of aging and progressive degeneration in the non-operated knee. Supporting this, previous studies have demonstrated that the condition of the contralateral limb significantly influences long-term outcomes following unilateral TKA [23].

Management of bilateral knee OA remains complex. While unilateral TKA can provide substantial symptomatic and functional improvement, it may not fully address the biomechanical imbalance associated with bilateral disease. Previous studies have shown that unilateral TKA can improve quality of life in patients with bilateral OA [24], although some patients may subsequently require contralateral intervention. Our findings are consistent with this observation, as patients experienced meaningful early functional gains following unilateral surgery.

Given the observed trend toward decline in balance function at later follow-up, clinicians should consider close monitoring of the contralateral knee. Predictors of subsequent contralateral TKA have been described in the literature [25], emphasizing the need for individualized treatment strategies to optimize long-term outcomes.

This study has several limitations that should be considered when interpreting the findings. First, its retrospective, single-center design precludes causal inference and may limit the generalizability of the results. Second, the relatively small sample size (n = 51) and the predominance of female patients may have introduced selection bias and further limited the external validity of the findings. Third, potential confounding factors, including progression of contralateral knee OA, variations in physical activity levels, and other musculoskeletal or medical comorbidities, were not systematically evaluated and may have influenced balance outcomes. Fourth, balance assessment was limited to the TUG and OLS tests. Although these are validated and widely used clinical measures of functional mobility and static balance, they do not comprehensively assess all aspects of postural control, and the inclusion of instrumented balance assessments or additional functional outcome measures may provide a more comprehensive evaluation in future studies. Finally, owing to the observational nature of this study, the deterioration in balance observed over time cannot be attributed solely to the progression of contralateral knee OA, and other unmeasured factors may also have contributed to the findings. Therefore, larger prospective, multicenter studies with comprehensive balance assessments are warranted to confirm these results and further elucidate the mechanisms underlying changes in balance following unilateral TKA in patients with bilateral knee OA.

Despite these limitations, this study provides clinically relevant insights into the temporal pattern and durability of balance recovery following unilateral TKA in patients with bilateral knee OA, which may assist clinicians in patient counselling and the planning of staged bilateral arthroplasty.

## Conclusions

Unilateral TKA in patients with bilateral knee OA was associated with significant short-term improvements in both static and dynamic balance. Although these improvements tended to decline at later follow-up, the underlying factors contributing to this change could not be determined in the present study. These findings suggest that the benefits of unilateral TKA on balance may diminish over time in some patients, underscoring the importance of comprehensive clinical evaluation and longitudinal follow-up. Larger prospective studies are warranted to identify the factors influencing long-term balance outcomes and to optimize the timing of staged bilateral TKA in appropriately selected patients.
